# Surgeon-performed ultrasound guided fine-needle aspirate biopsy with report of learning curve; a consecutive case-series study

**DOI:** 10.1186/s40463-015-0099-x

**Published:** 2015-10-28

**Authors:** Vinay T. Fernandes, Robert J. De Santis, Danny J. Enepekides, Kevin M. Higgins

**Affiliations:** Department of Otolaryngology-Head & Neck Surgery, University of Toronto, Toronto, Canada; Sunnybrook Health Sciences Centre, 2075 Bayview Avenue, Suite M1 102, Toronto, ON M4N 3 M5 Canada; Department of Otolaryngology–Head & Neck Surgery, University of Toronto, Sunnybrook Health Sciences Centre, 2075 Bayview Avenue, Suite M1 102, Toronto, ON M4N 3 M5 Canada

**Keywords:** Ultrasound-guided fine-needle aspiration biopsy, Surgeon preformed, Thyroid nodules, Cystic lesions, Microcalcifications, Learning curve, Adequacy

## Abstract

**Background:**

Fine-needle aspiration biopsy has become the standard of care for the evaluation of thyroid nodules. More recently, the use of ultrasound guided fine-needle aspiration biopsy (UG-FNAB) has improved adequacy of sampling. Now there has been improved access to UG-FNAB as ultrasound technology has become more accessible. Here we review the adequacy rate and learning curve of a single surgeon starting at the adoption of UG-FNAB into surgical practice.

**Methods:**

UG-FNABs performed at Sunnybrook Health Sciences Centre from 2010 to 2015 were reviewed retrospectively. Nodule characteristics were recorded along with cytopathology and final pathology reports. Chi-square analysis, followed by the reporting of odds ratios with confidence intervals, were used to assess the statistical significance and frequencies, respectively, of nodule characteristics amongst both diagnostic and non-diagnostic samples. A multiple regression analysis was conducted to determine if any nodule characteristic were predictive of adequacy of UG-FNABs. The learning curve was assessed by calculating the eventual adequacy rates across each year, and its statistical significance was measured using Fischer’s Exact Test.

**Results:**

In total 423 biopsies were reviewed in 289 patients. The average nodule size was 23.05 mm. When examining if each patient eventually received a diagnostic UG-FNAB, regardless of the number attempts, adequacy was seen to increase from 70.8 % in 2010 to, 81.0 % in 2011, 90.3 % in 2012, 85.7 % in 2013, 89.7 % in 2014, and 94.3 % in 2015 (Fischer’s Exact Test, *p* = 0.049). Cystic (χ^2^ = 19.70, *p* <0.001) nodules were found to yield higher rates of non-diagnostic samples, and their absence are predictive of obtaining an adequate biopsy as seen in a multiple regression analysis (*p* < 0.001) Adequacy of repeat biopsies following an initial non-diagnostic sample was 75.0 %.

**Conclusions:**

Surgeons are capable of performing UG-FNAB with a learning curve noted to achieve standard adequacy rates. Cystic nodules are shown to yield more non-diagnostic samples in the surgeon’s office.

## Background

Fine needle aspirates (FNA) are the standard of care for evaluating thyroid nodules. The use of FNA has decreased the percentage of surgical pathology specimens containing only benign thyroid tissue, and therefore decreased the amount of unnecessary operations. While historically these biopsies have been performed by palpation alone in the office, a shift toward biopsy under ultrasound guidance has increased the adequacy rate of biopsies to greater than 80 % [1–10]. With the increased availability of ultrasound technology in the office setting, head and neck surgeons and endocrinologists have begun incorporating the tool into their practice.

Office-based ultrasound and biopsy offers several potential benefits for patients. Although there is a paucity of evidence examining the patient experience, it is intuitive that patients who are able to undergo an ultrasound and biopsy at their surgeon’s office following recommendation will wait less than those being referred to another clinic for biopsy. Furthermore, patients undergoing surveillance of thyroid nodules often have anxiety relating to the lack of diagnosis with the nodule. Many patients speak of the comfort they feel having their primary clinician perform the ultrasound surveillance and biopsy rather than a technologist or outside physician with whom they have no ongoing clinical relationship. For those previously biopsied benign nodules of large size, the surgeon’s ability to survey them might reduce unnecessary surgery and further decrease the rate of benign pathology in surgical reports.

Previous studies have reported on the experience of radiologists as well as patient wait times for surgery and diagnostic accuracy for surgeon performed UG-FNABs. However, here we report the largest Canadian series of ultrasound-guided fine needle aspirate biopsies (UG-FNAB) conducted by surgeons.

## Methods

### Patients

Consecutive patient charts were reviewed from 2010 to 2015. Charts were collated from a list created by the Sunnybrook Health Sciences Centre (SHSC) otolaryngology office manager of patients catalogued as having undergone a thyroid biopsy with ultrasound guidance in the department. This search yielded 652 individual biopsies, which were then evaluated by a two reviewers. 229 charts were excluded: 38 had no ultrasound report available, 78 were not specifically thyroid biopsies, 28 were found to be duplicates, 39 had no cytology report available, and 46 were performed by a different practitioner. This project was approved by the Research Ethics Board of Sunnybrook Health Science Centre (ID 169–2013).

### Surgical technique

A Hitachi Aloka© ultrasound machine was used to perform a diagnostic ultrasound of both thyroid lobes and the lateral neck. The principal investigator conducted all UG-FNABs. Nodules with suspicious features were targeted according to ATA guidelines. The patient was placed in the supine position with a green towel placed for semi sterile technique. A 10 MHz probe identified the nodule, and using a 25 G needle nodules were targeted using primarily the capillary suction technique using multiple passes, and a combination of the short and long axis techniques.

### Data collection

Data extracted from patient charts included demographic information, FNA cytology reports and pathology reports. Adequacy of specimen and diagnosis was reported by the cytopathologist at SHSC according to the Bethesda system for reporting thyroid fine needle aspirate biopsy (FNAB). Nodule characteristics were drawn from the ultrasound procedure report.

### Analysis

Statistical analysis was completed with SPSS® (V20, IBM Corp ©). Statistical significance for all tests were set at *p* < 0.05. Chi-square analysis, followed by the reporting of odds ratios (OR) with confidence intervals (CI), was used to assess the statistical significance and measure of association, respectively, of nodule characteristics amongst both diagnostic and non-diagnostic samples. A multiple regression was conducted to determine if any nodule characteristic were predictive of adequacy of UG-FNABs. The learning curve was assessed by calculating the eventual adequacy rates across each year, and its statistical significance was measured using Fischer’s Exact Test. The outcomes were recorded starting from the time at which the practice of using UG-FNAB was adopted into clinical practice. A comprehensive review of the North American and European English language literature was performed with the assistance of SHSC Librarians.

## Results

**Fig. 1:**
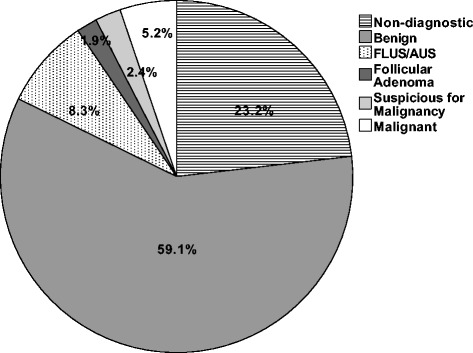
Fine needle aspirate biopsies by Bethesda classification. *Of all the biopsies taken with ultrasound guidance, 23.2 % yielded non-diagnostic specimens, 59.1 % benign, 8.3 % were either follicular lesion of undetermined significance or atypia of undetermined significance, 1.9 % follicular adenoma, 2.4 % suspicious for malignancy and 5.2 % reported as malignancy*

**Fig. 2:**
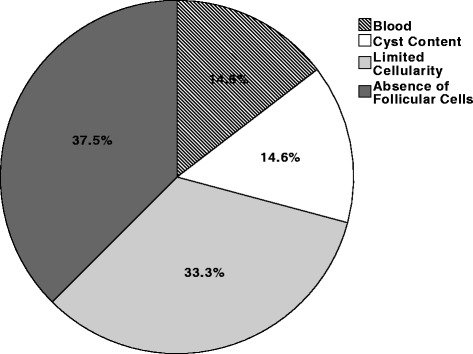
Breakdown of reason given for a specimen being non-diagnostic. *Of all the non-diagnostic biopsies, our cytopathologists commented that they were non-diagnostic for having blood (14.6 %), an absence of follicular cells (37.5 %), having cyst contents (14.6 %), or having limited cellularity (33.3 %)*

In total, 423 separate biopsies were examined in 289 individuals. Subject characteristics are noted in Table 1. The overall eventual adequacy rate was 86.8 %, which means that 86.8 % of the patients across all years, regardless of the number of biopsies, received a diagnostic UG-FNAB. Figure 1 outlines the breakdown of cytopathology reports according to Bethesda classification. Amongst the non-diagnostic specimens, pathologists reported that 14 (14.6 %) were due to blood, 14 (14.6 %) were due to cyst contents, 36 (37.5 %) had no follicular cells, and 32 (33.3 %) were reported as having limited cellularity to make a diagnosis (Fig. 2).

**Table 1:** Subject characteristics

Total patients *(n)*	289
Total UG-FNAB	423
Age	55 (20–89)
Gender (M:F)	77:212
Nodule Size	23.05 mm (3.9–100) σ = 14.04
Adequacy	86.9 %

**Fig. 3:**
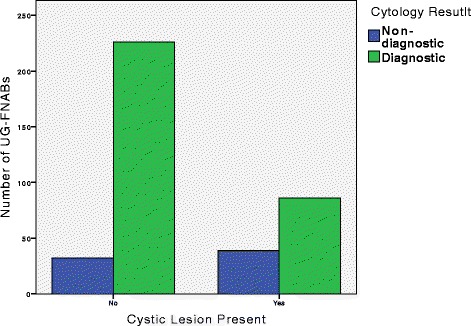
Distribution of diagnostic and non-diagnostic UG-FNABs by presence of cystic lesions. *This double bar graph represents the frequency of diagnostic and non-diagnostic UG-FNABs when cystic components are absent or present in the biopsied nodule. In total, 383 biopsies reported the cystic nature of the nodule; with 86 diagnostic and 39 non-diagnostic lesions with a cystic component; and 226 diagnostic and 32 non-diagnostic lesions without a cystic component*

There was no significant difference when adequacy was contrasted in terms of internal vascularity, echotecture, irregular margins, or microcalcifications in a chi-square analysis. However, cystic lesions (Fig. 3) (χ^2^ = 19.70, *p* < 0.001, OR = 3.21 CI: 1.89–5.43) were more commonly found amongst non-diagnostic specimens. Furthermore, a multivariate logistic regression was run to predict UG-FNAB adequacy from nodule size, cystic nature, echotexture, and the presence of microcalcifications. These variables statistically significantly predicted adequacy (χ^2^ = 15.71, *p* = 0.003, df = 4). However, Nagelkerke’s R^2^ of 0.091 shows that the independent variables were a poor model for prediction of adequacy. Moreoever, only one variable, cystic nature, added statistically significantly to the prediction, *p* < 0.001. The results from the multiple regression support those found in the chi-square analysis.

**Fig. 4:**
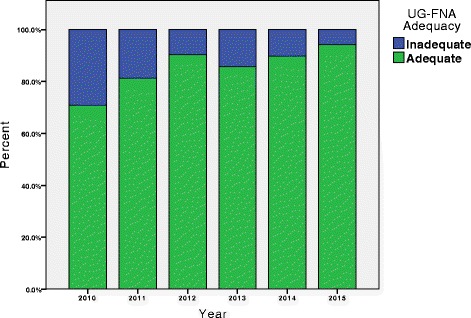
Eventual adequacy rate over time of all UG-FNABs divided by year. *This double bar graph represents the breakdown of patients who eventually received a diagnostic UG-FNAB, regardless of the number of biopsies, and those who never had a diagnostic UG-FNAB. The 289 patients were divided into the years they received their biopsy; with 17 diagnostic and 7 non-diagnostic in 2010; 47 diagnostic and 12 non-diagnostic in 2011; 56 diagnostic and 6 non-diagnostic in 2012; 30 diagnostic and 5 non-diagnostic in 2013; 35 diagnostic and 4 non-diagnostic in 2014; and 66 diagnostic and 4 non-diagnostic in 2015*

The learning curve was demonstrated sufficiently by measuring the proportion of patients per annum who eventually received a diagnostic UG-FNAB, regardless of the number attempts. Eventual adequacy (Fig. 4) was seen to increase from 70.8 % in 2010 to, 81.0 % in 2011, 90.3 % in 2012, 85.7 % in 2013, 89.7 % in 2014, and 94.3 % in 2015 (Fischer’s Exact Test, *p* = 0.032). Amongst the UG-FNABs that were repeat biopsies following an initial non-diagnostic sample, the subsequent adequacy rate was 75.0 %, with an average of 12.4 days (*n* = 16) between initial and repeat biopsies across all years.

## Discussion

A review of surgeon-performed biopsies at Sunnybrook Health Sciences Centre has demonstrated an overall eventual adequacy rate of 86.9 %, with a clear learning curve experienced as the eventual adequacy rate improves each year, peaking at approximately 94.0 % in 2015. Our findings are consistent with the Canadian literature, which supports the association of cystic nodules were associated with non-diagnostic specimens [11] and shows that adequacy rates can approach 90 % [12]. Larger surgeon audits report overall adequacy rates of 70–92 % [1–4], while reports of radiologists, endocrinologists, and pathologists performing UG-FNABs suggest adequacy rates varying from 67 % to 94 % [5–10]. The current adequacy rate in our practice is comparable to those available in the literature.

Our institution uses the Bethesda system to report thyroid cytopathology [13]. The evidence reviewed at the Bethesda meeting suggested that non-diagnostic rates are between 2 and 20 %. Our data demonstrate that over time the adequacy rate will approach acceptable rates in a surgeon’s office, showing evidence of a learning curve. To mitigate the impact of physician learning on patient safety early on, patients with unsatisfactory biopsies were sent to radiology for repeat biopsy. As time went on and non-diagnostic rates diminished, this practice was abandoned. A review of repeat biopsies following non-diagnostic specimens in our centre demonstrated improved adequacy up to 75.0 % overall with a second biopsy. These figures are important, as the common practice to send a patient for a radiologist performed biopsy might increase the rate of lost-to follow up following non-diagnostic sampling, and certainly increases wait times. At our institution, rather than wait 4–6 weeks for a radiologist appointment, patients have the benefit of immediate biopsy at clinic visit. If repeat biopsy is sought during a clinic visit in which non-diagnostic results are reviewed, that repeat biopsy can be performed the same day.

Surgeon-performed ultrasound-guided fine needle aspirates has been adopted over the past decade in North America. As more and more physicians adopt the practice, it is necessary to obtain quality measures to ensure the procedure is performed adequately. One measure that must be understood in the adoption of a new procedure is the learning curve. The concept comes from the engineering industry; Wright described improving efficiency of production in his 1930′s thesis as the skill of the workers increased [14]. While this concept is intuitive now with UG-FNABs, the number of biopsies needed to attain acceptable adequacy rates remains poorly answered. A South Korean comparison of an “experienced” versus “less-experienced” radiologist suggested no significant difference in adequacy rates to achieve 94 % [15]. However, not only had their experienced radiologist performed over 12,000 biopsies, their less experienced one had already performed at least 500. Mahoney reported astounding inadequacy rates of 0.4 % of thyroid biopsies, but admitted that they were within a 3-year interval after 15 years of experience [16].

In our assessment of learning curve, we analyzed adequacy within each calendar year, which resulted in a meaningful comparison. Published descriptions of UG-FNAB learning curve involve setting 100 patients [17], and similar case numbers have been described for the learning curve of other medical procedures. Surgical procedures are somewhat different, and many authors describe somewhere between 20 and 50 cases to achieve competency [18]. CT-guided FNA has been suggested to have a learning curve of only 40 cases, with DelGaudio reporting an improvement from 80 to 94 % adequacy [19].

A large proportion of the nodules biopsied had some portion of cystic content within them. The American Thyroid Association guidelines suggest that there is no indication for the biopsy of purely cystic nodules. In our series, forty-five of the non-diagnostic specimens contained large cyst content. Some purely cystic nodules were aspirated for causing compression symptoms causing an overall adequacy rate for nodules described as cystic of 65.9 %. These aspirates recorded as cystic include large spongiform nodules, and so all were included in our non-diagnostic category. Controversy exists as to whether cystic content should be placed into a separate category on its own. In our study, cystic lesions were seen to more likely yield non-diagnostic results, likely because they have little to no follicular cells necessary for diagnosis. This relationship can help to explain why we have a less than perfect adequacy rate with UG-FNAB, even after a physician achieves expertise. Additionally, nodules with microcalcifications were more frequently observed in specimens that were diagnostic. Since the presence of microcalcifications is associated with papillary thyroid carcinoma, the above relationship reinforces that malignant nodules are generally being captured by UG-FNAB.

Once an audit of practice is performed, the next question becomes how to improve quality. One method is to standardize the procedure, with which some authors have reported success [7]. Sidiropoulous reported improved adequacy following standardization of technique (needle size and number of passes), preparation of samples including both slide prep and liquid preparation (which is quite time consuming), and personnel (difficult in a teaching institution where residents and fellows are involved). There is further evidence that standardizing the number of passes improves adequacy, as Naim 2013 showed inadequacy rates of 33.8 %, 23.4 %, and 13.7 % with 1, 2 and 3 needle passes respectively.

The utilization of on-site cytopathology is the subject of controversy in the literature. Reports with on-site evaluation approach 95 % [20, 21]. Some comparisons report increase in diagnostic rates from 73 % to over 90 % [22] once the process is implemented. Olson showed that both cytopathologists and less expensive technologists are able to achieve similar levels of on-site evaluation of adequacy [23]. The issue of course with implementation is cost. Eedes calculated that in Boston, the cytopathologist would spend 220 min of time for each additional diagnostic case [24]. Zanocco performed a formal cost-effectiveness analysis using a decision model to compare strategies in the U.S. health system. He found that when the adequacy rate without on-site evaluation is less than 85 %, on-site evaluation became cost-effective [25].

This review has several limitations owing to design. Without prospectively collected data, factors such as surgical technique, amount of passes, and recording of nodule characteristics on ultrasound reports will be variable. One of the main challenges faced was the lack of consistent reporting of characteristics, which made analysis difficult. We did not have consistent data regarding the specific indication for biopsy. Future audits will include prospectively collected data using standardized forms. Although complications were not specifically recorded in this database, to the author’s knowledge there were no complications.

## Conclusion

Analysis of nodules’ characteristics revealed that cystic lesions are more likely to yield non-diagnostic sampling, which can help to explain why we have a less than perfect adequacy rate with UG-FNAB, even after a physician achieves expertise. However, this report ultimately concluded that surgeon performed ultrasound guided FNA of the thyroid is a useful tool that can be implemented in the head and neck surgeon’s office with ease.

## Abbreviations

FNA: Fine needle aspirate
UG-FNAB: Surgeon preformed ultrasound-guided fine needle aspirate biopsy
SHSC: Sunnybrook health sciences centre
FNAB: Fine needle aspirates biopsy
OR: Odds ratio
CI: Confidence interval
